# Hijacking the Hexosamine Biosynthetic Pathway to Promote EMT-Mediated Neoplastic Phenotypes

**DOI:** 10.3389/fonc.2016.00085

**Published:** 2016-04-18

**Authors:** Kekoa Taparra, Phuoc T. Tran, Natasha E. Zachara

**Affiliations:** ^1^Department of Radiation Oncology and Molecular Radiation Sciences, Sidney Kimmel Comprehensive Cancer Center, Johns Hopkins University School of Medicine, Baltimore, MD, USA; ^2^Program in Cellular and Molecular Medicine, Johns Hopkins University School of Medicine, Baltimore, MD, USA; ^3^Department of Oncology, Sidney Kimmel Comprehensive Cancer Center, Johns Hopkins University School of Medicine, Baltimore, MD, USA; ^4^Department of Urology, Johns Hopkins University School of Medicine, Baltimore, MD, USA; ^5^Department of Biological Chemistry, Johns Hopkins University School of Medicine, Baltimore, MD, USA

**Keywords:** glycoproteins, glycosylation, *O*-GlcNAcylation, *O*-GlcNAc, EMT, cancer, nucleotide sugar, metabolism

## Abstract

The epithelial–mesenchymal transition (EMT) is a highly conserved program necessary for orchestrating distant cell migration during embryonic development. Multiple studies in cancer have demonstrated a critical role for EMT during the initial stages of tumorigenesis and later during tumor invasion. Transcription factors (TFs) such as SNAIL, TWIST, and ZEB are master EMT regulators that are aberrantly overexpressed in many malignancies. Recent evidence correlates EMT-related transcriptomic alterations with metabolic reprograming in cancer. Metabolic alterations may allow cancer to adapt to environmental stressors, supporting the irregular macromolecular demand of rapid proliferation. One potential metabolic pathway of increasing importance is the hexosamine biosynthesis pathway (HBP). The HBP utilizes glycolytic intermediates to generate the metabolite UDP–GlcNAc. This and other charged nucleotide sugars serve as the basis for biosynthesis of glycoproteins and other glycoconjugates. Recent reports in the field of glycobiology have cultivated great curiosity within the cancer research community. However, specific mechanistic relationships between the HBP and fundamental pathways of cancer, such as EMT, have yet to be elucidated. Altered protein glycosylation downstream of the HBP is well positioned to mediate many cellular changes associated with EMT including cell–cell adhesion, responsiveness to growth factors, immune system evasion, and signal transduction programs. Here, we outline some of the basics of the HBP and putative roles the HBP may have in driving EMT-related cancer processes. With novel appreciation of the HBP’s connection to EMT, we hope to illuminate the potential for new therapeutic targets of cancer.

## Introduction

Since the time of Otto Warburg in the 1930s, scientists have been intrigued by the unique metabolic profile of cancer cells (1, 2). Current research corroborates Warburg’s original observation that cancer prefers glycolysis over mitochondrial oxidative phosphorylation (OXPHOS) (3). Initially, this metabolic reprograming appeared paradoxical due to the inefficiencies of glycolysis (i.e., ~38 ATP from OXPHOS versus 2 ATP from glycolysis). Despite early conflicting viewpoints on the Warburg Effect, aerobic glycolysis stands at the center of cancer metabolism demonstrating its importance as an “Emerging Hallmark of Cancer” (4).

Despite decades of research, the molecular advantages of the Warburg effect in cancer are still being interrogated (5). One popular explanation is the “Glycolytic Intermediate Diversion” hypothesis (6, 7). This hypothesis suggests that glycolysis is well positioned to support anabolic cell growth as it provides the metabolic intermediates (e.g., nucleosides, amino acids, and other carbon compounds) necessary for enzymatic reactions and organelle assembly. A second hypothesis involves the notion of “Cell Subpopulations” (8–10). This hypothesis posits that lactate from “Warburg-effect cells” is sent to neighboring cells, which utilize lactate through the citric acid cycle. The cell subpopulations symbiotically trade off waste for energy to support cancer progression. Interestingly, both hypotheses demonstrate the ability of the neoplastic state to commandeer normal biological processes observed in development and normal physiology (4).

The energetic demand required to survive adverse tumor environments is likely only a fraction of the functional significance underlying cancer metabolic reprograming. It is likely that glycolytic byproducts reinforce the cancer phenotype by modulating not just metabolic maintenance but also altering other cellular structures and functions. In particular, the role of post-translational modifications (PTM), such as glycosylation, are becoming of increasing importance as they provide rapid, reversible adaptations to the stressors that occur during early tumorigenesis. Recent studies have revealed new potential cancer treatment strategies specifically targeting these glycoconjugates (11).

Interestingly, one metabolic pathway with the potential of impacting functional macromolecular structures in cancer is an understudied pathway called the “hexosamine biosynthetic pathway” (HBP) (12–15). One downstream metabolite of this pathway, uridine diphosphate–*N*-acetylglucosamine (UDP–GlcNAc), serves as an essential building block for glycoconjugate biosynthesis. This pathway is well positioned to not only affect metabolic intermediates but also functional glycans that accelerate cancer progression (11, 16). The HBP has only recently gained traction in cancer biology and is becoming of increasing importance (17).

The epithelial–mesenchymal transition (EMT) is a conserved epithelial plasticity program capable of impacting cellular morphology, migration, stem cell-ness, among other malignant phenotypes (18). Moreover, the EMT is involved throughout the natural history of cancer from tumorigenesis to late metastatic progression (19–21). Master transcriptional regulators of EMT (i.e., TWIST, SNAIL, and ZEB) are elevated in a wide range of primary and metastatic tumors. Recent evidence demonstrates that the expression of key enzymes in the HBP is upregulated in cancer cells with a mesenchymal phenotype (22). Thus, in this review, we will highlight some of the relevant glycoconjugates downstream of the HBP and the implications this has on EMT-mediated cancer programs.

## The Epithelial–Mesenchymal Transition

**Figure 1:**
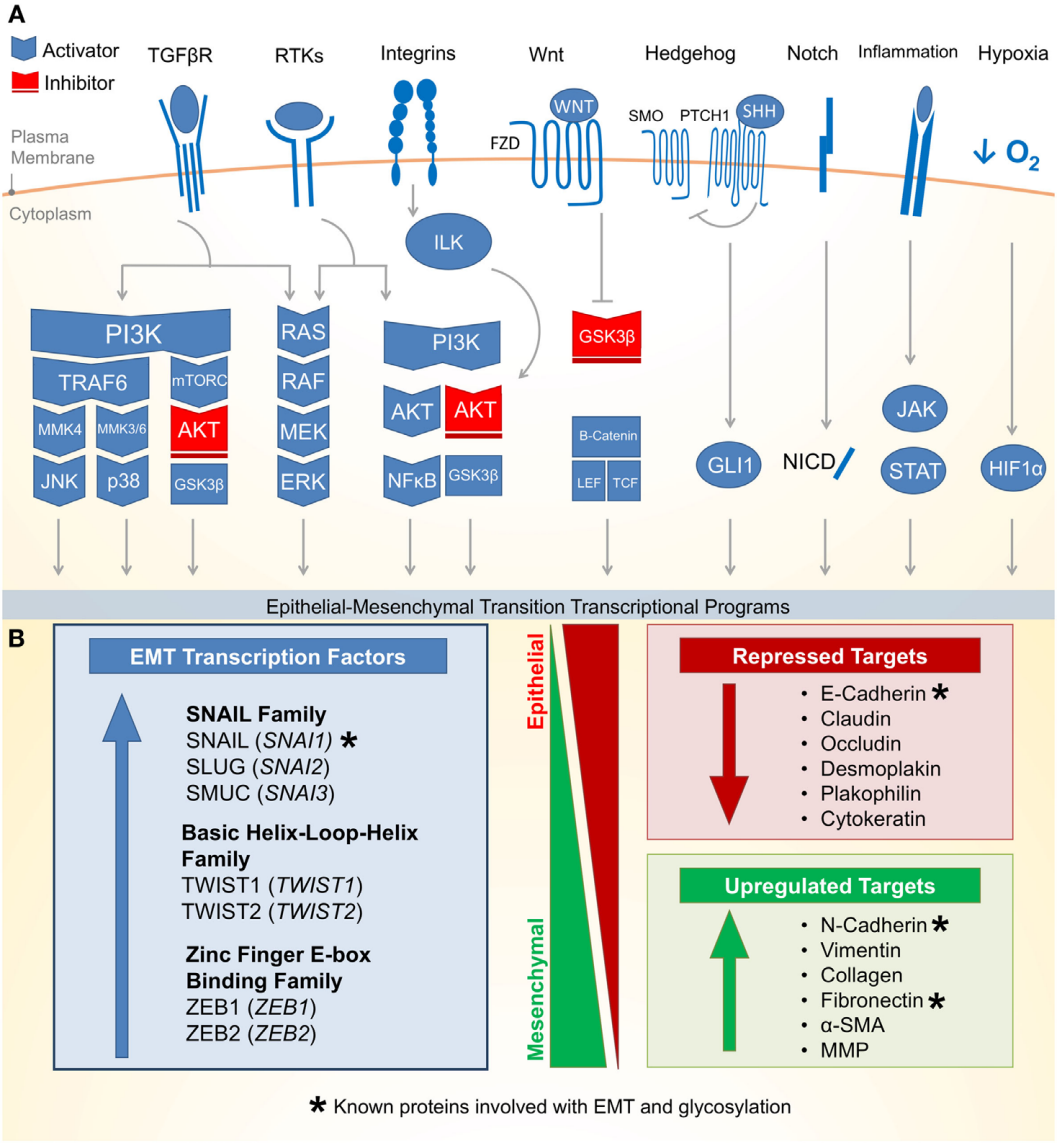
**Molecular pathways and targets of the epithelial–mesenchymal transition**. **(A)** (1) One of the most well characterized EMT-inducing pathways is the transforming growth factor-β (TGF-β) family receptors capable of inducing PI3K–AKT, ERK MAPK, p38 MAPK, and JUN N-terminal kinase (JNK) pathways (activating pathways in blue; inactivating pathways in red). (2) The RAS–RAF–MEK–ERK MAPK pathway lies downstream of a number of growth factor activated receptor tyrosine kinases (RTKs) and activates a number of major EMT transcription factors (TFs). (3) Integrin signaling can have a multipronged effect on EMT by both interrupting critical epithelial adhesion molecules (e.g., E-cadherin) and antagonizing GSK3-β via the integrin-linked kinase (ILK)-AKT signaling, thus promoting EMT. (4) WNT signaling can also interfere with GSK3-β, thus stabilizing β-catenin to promote EMT transcriptional programs in cooperation with lymphoid enhancer-binding factor 1 (LEF1) and T-cell factor (TCF). (5) The Hedgehog (HH)-glioma 1 (GLI1) and (6) NOTCH pathway both can promote transcription of the EMT regulators. (7) Recently, a number of inflammatory pathways downstream of interleukin (IL) signaling (e.g., IL-6) have demonstrated the activation of the Janus-kinase (JAK)-signal transducer and activator of transcription 3 (STAT3) pathway, which in turn promotes EMT transcription factors. (8) Hypoxia is capable of activating a number of key components of EMT through the hypoxia-induced factor 1 (HIF1α). **(B)** Downstream of these signal transduction pathways leading to EMT are a variety of transcription factors with the ability to alter epithelial gene expression. As an epithelial plasticity program, many of the target genes altered include adhesion molecules. Known glycosylated proteins involved with EMT are denoted with an asterisk (*).

Epithelial–mesenchymal transition is an essential epithelial plasticity program deployed during development (23, 24), wound healing (25–27), and stem cell maintenance (28–31). The major characteristics of EMT include loss of cellular adhesion, reorganization of cytoskeleton, loss of cellular polarity, and a switch from epithelial to mesenchymal gene expression (18). Many of these EMT pathways are activated by extracellular signaling, highlighting the importance of the tumor microenvironment for the induction of EMT. Figure 1A outlines eight critical EMT-activating pathways: TGF-β, receptor tyrosine kinases (RTKs), integrin, WNT, NOTCH, Hedgehog (HH), hypoxia inducible factor 1α (HIF1α), and JAK/STAT.

There are three major families of transcription factors (TFs) that contribute to EMT and may also be general drivers of cancer (Figure 1B): (1) the zinc finger protein SNAIL family (SNAI1, SNAI2, and SNAI3) (32), (2) the basic Helix-Loop-Helix (bHLH) proteins TWIST1 and TWIST2 (33), and (3) the zinc-finger E-box binding (ZEB) family of TFs (34). These TFs are evolutionarily conserved and critical for development. They bind short DNA segments called enhancer boxes (E-boxes) with the consensus sequence “CANNTG.” Like many TFs, they are able to modulate transcription by recruiting a variety of epigenetic regulators to alter the chromatin landscape of epithelial plasticity genes and interactions with transcriptional coactivators and corepressors (35).

The most well-established gene targets of EMT TFs are generally involved in epithelial cell adhesion (36–38). Cadherins represent a family of calcium-dependent cell–cell adhesion proteins particularly targeted by EMT (39–41). Loss of epithelial cadherin (E-cadherin) is a major hallmark of EMT (42–44). Thus, loss of E-cadherin has been used as a biomarker for many cancers. Additionally, loss of tight junctions (e.g., claudin and occludin), desmosomes (e.g., desmoplakin and plakophilin), and cytokeratins (intermediate filaments) are commonly observed during EMT (18). Conversely, while epithelial markers are repressed, mesenchymal markers are increased during EMT. These markers include N-cadherin, vimentin, and fibronectin (18). Following the transcriptional alterations of these adhesion molecules, protein degradation and endocytosis aid in recycling epithelial adhesion molecules to promote progress through EMT (45).

Altered gene expression of EMT targets, such as those involved in cellular adhesion, often facilitate biological and pathological functions such as migration and invasion (46–48). Upon detaching from the basal epithelium, epithelial cells undergoing EMT may alter their extracellular environment by expressing matrix metalloproteinases (MMPs) to promote directional migration (49–51). During migration, adhesion molecules are disproportionately redistributed between the leading and trailing edge of the cell, which allows the cell to coordinate directed migration leading to tumor dissemination and metastasis (24, 52). Beyond metastasis, EMT has recently been attributed to more fundamental roles in cancer biology including suppressing apoptosis and senescence (53). The EMT has also been implicated in immune evasion (54) and metabolic reprograming (22, 55) of cancer cells. Together, the data discussed above suggest that the EMT program promotes many cancer cell phenotypes leading to malignancy.

## The Hexosamine Biosynthetic Pathway

**Figure 2:**
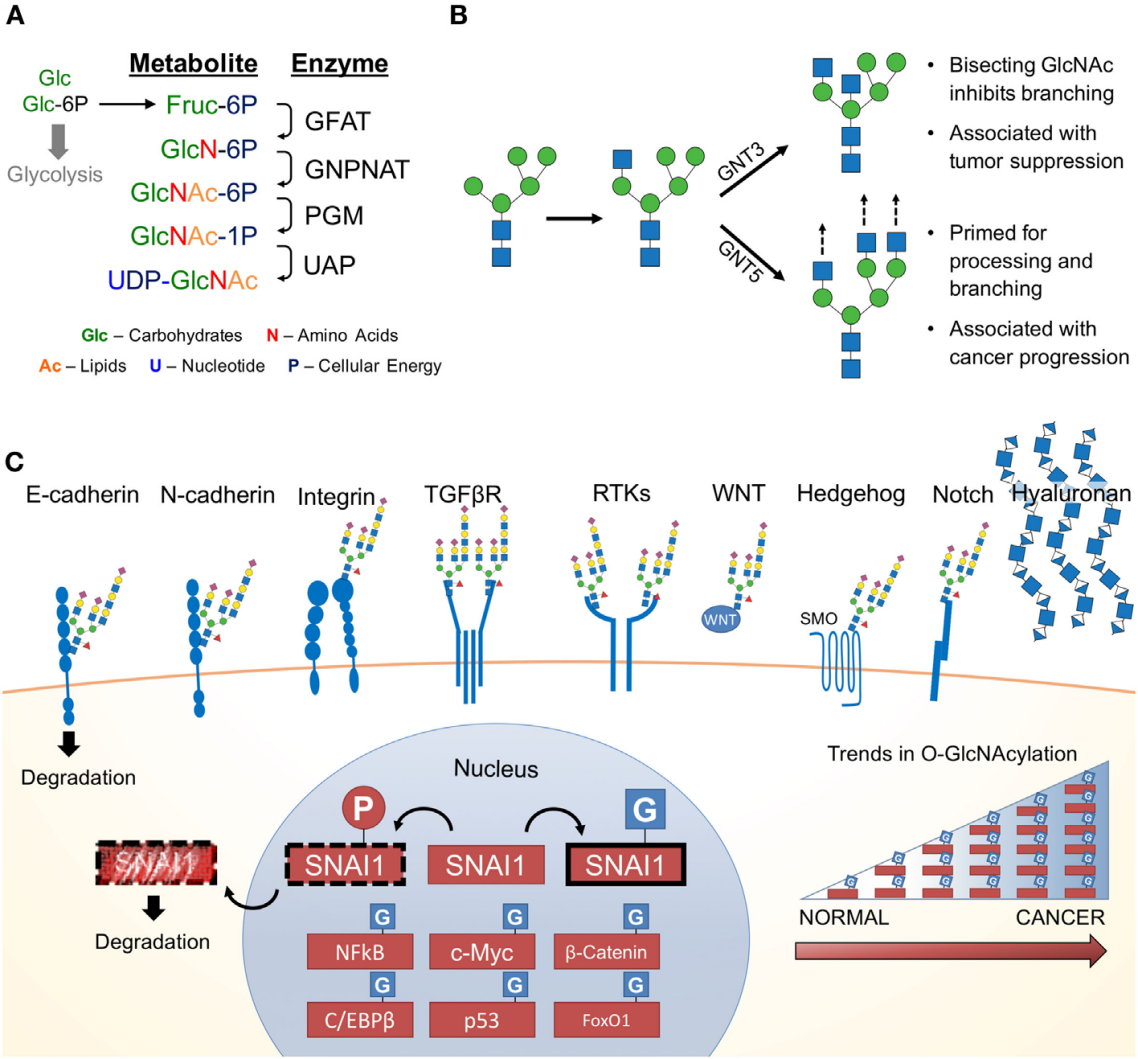
**The hexosamine biosynthetic pathway (HBP) and glycosylated EMT targets**. **(A)** First, the rate limiting enzyme of the HBP, glutamine:fructose-6-phosphate transaminase (GFAT), uses glutamine (Gln) as an amine donor to convert Fru-6P into glucosamine-6-P (GlcN-6P). Second, glucosamine-phosphate *N*-acetyltransferase (GNPNAT) *N*-acetylates GlcN-6P in an acetyl-CoA-mediated reaction to form *N*-acetylglucosamine-6-P (GlcNAc-6P). Third, phosphoglucomutase (*PGM*) isomerizes GlcNAc-6P to the highly active GlcNAc-1P. The final step is catalyzed by UDP–*N*-acetylglucosamine pyrophosphorylase (*UAP1*) and charges GlcNAc-1P with UDP to form uridine-5′-diphosphate-*N*-acetylglucosamine (UDP–GlcNAc). **(B)** UDP–GlcNAc (depicted as a blue square) is essential for N-glycosylation processing and elongation. One critical pivot point includes the branching of complex *N*-glycans. Inhibiting this process with a bisecting GlcNAc is associated with tumor suppressive phenotypes. In contrast, cancers have aberrant expression of glycosyltransferases responsible for branching and elongating complex *N*-glycans. **(C)** Many of the proteins commonly associated with promoting EMT are modified by glycans containing GlcNAc and are found on the cell surface. Hyaluronan, a glycosaminoglycan, is also found extracellularly and is a polymer of glucuronic acid and *N*-acetylglucosamine. Many nuclear, cytoplasmic and mitochondrial proteins are modified by monosaccharides of *O*-linked *N*-acetylglucosamine (*O*-GlcNAc), including many transcription factors, which appear to be stabilized by glycosylation (63). Numerous studies have identified various cancers with elevated levels of pan-*O*-GlcNAcylation (64).

Since the 1950s, cancer has been notorious for its addiction to glucose and glutamine (7, 56–58). Upon depletion of these carbon sources in cancer cell culture media, cellular growth is abrogated. Both glucose and glutamine (Gln) are essential for the first committed step and rate-limiting step of the HBP, the conversion of fructose-6-phosphate (Fru-6P) to glucosamine-6-phosphate. Approximately 2–5% of glucose (in adipocytes) is shunted through the HBP (59). Demonstrating the importance of extracellular glucose concentrations on the HBP, glucose starvation reduces UDP–GlcNAc levels (60, 61). Conversely, elevating extracellular glucose concentrations results in increased flux through the HBP (62). Figure 2A summarizes the four key enzymatic steps of the HBP:

(1) Glutamine:fructose-6-phosphate transaminase (GFAT; *GFPT*) utilizes glutamine in a transamination reaction, which converts fructose-6-phosphate (Fru-6-P) to glucosamine-6P (GlcN-6P);
(2) GlcN-6P is converted to *N*-acetylglucosamine-6-P (GlcNAc-6P) by Glucosamine-phosphate *N*-acetyltransferase (GNPNAT; *GNPNAT*), which requires acetyl-CoA;
(3) Phosphoglucomutase (PGM; *PGM*) isomerizes GlcNAc-6P to *N*-acetylglucosamine-1-phosphate (GlcNAc-1P);
(4) UDP–*N*-acetylglucosamine pyrophosphorylase (UAP1; *UAP1*) charges GlcNAc-1P with UDP to form uridine-5′-diphosphate–*N*-acetylglucosamine (UDP–GlcNAc).

Together, the four enzymes of the HBP orchestrate the *de novo* biosynthesis of the charged nucleotide sugar UDP–GlcNAc from glucose. This process can be manipulated by endogenous metabolites (i.e., glutamine) (65) as well as exogenous sugars (i.e., glucose, glucosamine, and *N*-acetylglucosamine) (66). Interestingly, this pathway is well positioned to sense the four macromolecules of life, coordinating carbohydrate, amino acid, lipid, and nucleotide donors through by Fru-6P, Gln, acetyl-CoA, and uridine, respectively (67). Despite the limited flux through the HBP, cellular UDP–GlcNAc levels can reach over 1 mM making it one of the most abundant high-energy cellular compounds (68). UDP–GlcNAc is utilized in the synthesis of numerous glycoconjugates and is interconverted into other nucleotide sugars (e.g., UDP–GalNAc, *N*-acetylmannosamine, CMP-neuraminic acid), which are incorporated into glycoconjugates (69). Together, the glycan structures downstream of the HBP metabolite, UDP–GlcNAc, influence a wide range of functional targets highly relevant to cancer and EMT.

Reinforcing the importance of UDP–GlcNAc incorporation, recent data suggest that the expression of multiple enzymes of the HBP and glycosyltransferases are altered in cancer, correlating with EMT, cancer progression, and metastasis. In a recent analysis using unsupervised hierarchical clustering of 1,704 metabolic genes and nearly 1,000 cancer cell lines, Shaul and colleagues identified a “mesenchymal metabolic signature” (MMS) (22). In this MMS, both *GFPT2* and *UAP1*, key enzymes in the HBP, were found to be essential for the mesenchymal phenotype (22, 70). In other studies, metabolites of the HBP (e.g., UDP–GlcNAc) were reported to be elevated in cancer cells and this was linked to survival (60).

Glycosyltransferases consistently elevated in multiple cancers (e.g., stomach and pancreas cancer) include β-1,4-mannosyl-glycoprotein 4-β-*N*-acetylglucosaminyltransferase (GNT3), α-1,6-mannosylglycoprotein 6-β-*N*-acetylglucosaminyltransferase A (GNT5), core 2 β-1,3-galactosyl-*O*-glycosyl-glycoprotein β-1,6-*N*-acetylglucosaminyltransferase (Core 2 GNT; GCNT1), *N*-acetyllactosaminide β-1,6-*N*-acetylglucosaminyl-transferase-isoform A (GCNT2), and UDP-*N*-acetylglucosamine-dolichyl-phosphate *N*-acetylglucosaminephosphotransferase (GPT1), encoded by the genes *MGAT3*, *MGAT5*, *GCNT1*, *GCNT2*, and *DPAGT1*, respectively (71). Notably, GNT5 is highly associated with breast, lung, and colon cancer metastasis (72–77), whereas GNT3 is associated with breast, skin, and colon cancer tumor suppression (78–80). GNT5 and GNT3 have antagonistic roles; GNT5 promotes complex *N*-linked glycan branching, whereas GNT3 suppresses branching (Figure 2B). It is thought that changes in glucose flux through the HBP impacts the function of GNT3 and GNT5 (66). The rate-limiting enzyme that forms the precursor for N-glycosylation, GPT1, has also been shown to drive proliferation, EMT, and cell morphology (81). As discussed below, flux through the HBP can alter the distribution patterns of glycosylation. To date, this has not been specifically studied in EMT. However, there are a number of glycoconjugates affected by changes in UDP–GlcNAc availability or changes in their biosynthesis. These glycoconjuagtes and their impact on EMT are discussed below.

## External GlcNAc-Containing Glycoconjugates Observed during EMT

Accumulating evidence strongly suggest changes in protein glycosylation impact numerous cancers including melanoma (82), pancreas (83, 84), colon (85), ovarian and breast (86), brain and lung (87), liver (88), and prostate (89) cancers. Generally, alterations in *N*-glycan structure profoundly affect cellular adhesion and epithelial morphology *in vitro* (90). Figures 2B,C show that many glycoproteins utilizing UDP–GlcNAc in their biosynthesis occur on key EMT adhesion molecules (e.g., E- and N-cadherin). E-cadherin has four putative *N*-linked glycosylation sites (91), which are modified by complex *N*-linked glycans. The number of “antennae” on these glycans is regulated by the competing activities of GNT3 and GNT5. The introduction of a bisecting GlcNAc by GNT3 (Figure 2B) reduces the number of antennae and thus complexity of the *N*-linked glycans. Epigenetic regulation of the gene encoding GNT3, *MGAT3*, stabilizes E-cadherin and inhibits EMT (92). In contrast, elevated activity or expression of GNT5 results in more complex *N*-glycans, which impairs E-cadherin localization and cellular aggregation in mice (93). Additional studies in mice have revealed that *MGAT5* knockdown leads to a reduction of *N*-glycosylated E-cadherin, which increases E-cadherin cis-dimerization, catenin recruitment, and cell membrane localization (94, 95). Importantly, aberrantly *N*-glycosyated E-cadherin is found in gastric cancer patients and correlates with poor patient survival (94).

Mesenchymal N-cadherin is also modified by *N*-linked glycans, and the modification of these glycans with GlcNAc by GNT5 promotes cell migration, MAPK signaling, and reduced adhesion (96, 97). Furthermore, N-cadherin *N*-glycans attract galectin-3, forming highly organized lipid rafts on the cell surface, which stabilizes the galectin lattice and enhances cancer cell mobility (98). This galectin lattice structure also recruits several major signaling receptors such as epidermal growth factor (EGF) receptor (EGFR) and TGF-β to promote oncogenic signaling (99, 100).

Integrins are heterodimeric glycoproteins responsible for cell–cell and cell–extracellular matrix interactions (101). The α5β1 integrin serves as the receptor for fibronectin, and their interaction is critical for cellular migration in development (102–104). While both integrin and fibronectin are *N*-glycosylated, the activity of GNT3 is associated with shorter less complex *N*-glycans, which is thought to result in reduced integrin-mediated EMT signaling (105, 106). Additionally, without *N*-linked glycans, integrins show significantly decreased heterodimerization, cell surface localization, and promotion of migration *in vitro* (107).

Receptor tyrosine kinases are vital to transducing external stimuli into internal signals for induction of EMT in many cancer (e.g., carcinomas). Interestingly, RTKs involved in growth and proliferation (e.g., EGFR) have approximately five times more *N*-glycosylation sites than receptors involved with organogenesis, differentiation, or cell cycle arrest (108). The HBP has been shown to drive changes in EGFR *N*-glycosylation; feeding both GNT5 wild type and GNT5 null tumor cells with *N*-acetylglucosamine elevated UDP–GlcNAc levels and the number of terminal GlcNAc residues on cell surface proteins. Analysis of the *N*-linked glycans demonstrated increased flux through the HBP results in increased triantennary structures in GNT5 null cells (twofold) and a smaller increase in both tri- and tetra-antennary *N*-glycans in GNT5 wild-type cells. Functionally, increased flux through the HBP-altered EGFR plasma membrane retention, active conformation, EGF ligand binding, and inhibition of endocytosis mediated degradation (109, 110). *N*-glycosylated EGFR recruits *N*-glycosylated TGF-βR to the galectin lattice thereby promoting TGF-β and SMAD autocrine signaling. TGF-βR with highly branched glycans, a result of increased GNT5 activity, localizes to the plasma membrane, binds galectin-3, inhibits receptor endocytosis, enhances TGF-βR heterodimerization, increases tumor metastasis, and promotes EMT-mediated cell migration (100, 111). TGF-β itself upregulates GCNT1, a critical GlcNAc branching enzyme, producing similar effects in prostate, colorectal, pancreatic, testicular, and breast cancers (112).

The WNT, NOTCH, and HH pathways are also critical for EMT and are modified by glycans that utilize GlcNAc for modulation of pathway activity. All 19 known WNT ligands contain at least one N-linked glycosylation site, and these sites are critical for ligand maturation, lipid processing, secretion, and β-catenin signal transduction (113, 114). WNT also regulates transcription of *DPAGT1* to promote EMT through E-cadherin glycosylation (81).

The Notch signaling pathway regulates cell proliferation, survival, and differentiation while glycosylation of components in this pathway are associated with poor prognosis and metastasis in numerous cancers (115, 116). Over two decades of research demonstrates the extracellular domain of Notch receptor is glycosylated with *N*-linked (117), *O*-fucose (117, 118), *O*-GlcNAc (119), and *O*-glucose (117, 120) glycans. Extension of *O*-fucose with GlcNAc [catalyzed by *O*-fucosylpeptide 3-beta-*N*-acetylglucosaminyltransferase (Fringe in *Drosophila*)] alters Notch ligand–receptor specificity. In *Drosophila*, extended *O*-fucose glycans are associated with increase sensitization of Notch to the Delta ligands and reduced sensitivity to the Serrate/Jagged ligands (116). Little is known about the impact of altered HBP flux on the Notch receptor, although one might postulate that changes in UDP–GlcNAc levels may alter Notch glycosylation and thus signaling downstream of this receptor. In the Sonic HH pathway, the G protein-couple receptor (GPCR), smoothened (SMO), is activated to promote cell proliferation and migration (121). Recently, critical *N*-glycans on SMO were found to abrogate HH induced cell migration due to blunted small heterotrimeric G_αi_ protein signaling (122).

Beyond the suite of GlcNAc-modified adhesion molecules and receptors, hyaluronic acid (hyaluronan or HA) is an oligomer found ubiquitously in the extracellular space particularly of connective, epithelial, and neural tissues (123). Human HA is a massive (0.5–2 MDa), unbranched glycosaminoglycan composed of the repeating disaccharide consisting of GlcNAc and glucuronic Acid (GlcNAcβ1–4GlcAβ1–3) (124). It is synthesized by HA synthase (HAS) and is extruded through the plasma membrane as it is synthesized. Recent reports suggest hyaluronan synthesis and catabolism is controlled by UDP–GlcNAc concentrations, with hyaluronan serving as a sink for excess UDP–GlcNAc (125). Recent studies have demonstrated that modulating levels of UDP–GlcNAc and glucuronic acid alter the localization of the HAS enzymes (126). Low levels of UDP–GlcNAc are associated with an inhibition of HA synthesis, whereas elevated levels of UDP–GlcNAc are associated with HA synthesis and melanoma progression (126). Consistent with these data, several studies have demonstrated patients with higher extracellular HA or HAS expression have a worse prognosis and survival with more aggressive and metastatic cancers including breast (127–129), prostate (130, 131), lung (132, 133), pancreatic (134), colorectal (135), and ovarian (136) cancers. With respect to EMT, high levels of HA are sufficient to induce the EMT in kidney and mammary epithelial cells (137). Taken together, HA synthesis is in part driven by the HBP, has been associated with EMT, and is found at high levels in many cancers.

## Nuclear, Cytoplasmic, and Mitochondrial Glycosylation Observed during EMT

Uridine diphosphate–*N*-acetylglucosamine can also be utilized for the synthesis of *O*-linked β-*N*-acetylglucosamine (*O*-GlcNAc), an essential PTM of metazoans (138). *O*-GlcNAc is found on more than 3,000 cytoplasmic, nuclear, and mitochondrial proteins (67). *O*-GlcNAcylation is thought to regulate protein function in a manner analogous to phosphorylation. *O*-GlcNAc has been demonstrated to regulate cellular processes such as epigenetics, transcription, translation, protein degradation, metabolism, ribosomal bioenergentics, and cytokinesis (139).

Unlike *N*-glycans, the *O*-GlcNAc modification (or *O*-GlcNAcylation) consists of a monosaccharide of GlcNAc covalently attached to serine and threonine residues through an *O*-glycosidic bond (138). Where *N*-linked glycan synthesis and processing is regulated by upwards of 18 enzymes (depending on the structure formed), the dynamic cycling of *O*-GlcNAc on proteins is regulated by just two enzymes: the *O*-GlcNAc transferase (OGT) and the *O*-GlcNAcase (OGA), which add and remove *O*-GlcNAc, respectively (140). OGT activity and substrate specificity are regulated by changes in UDP–GlcNAc concentrations, and this has led many to suggest that OGT may regulate cell function in a manner dependent on extracellular glucose concentrations (140). Cancer cells which are dependent on glucose and glutamine have been demonstrated to have high UDP–GlcNAc levels (*discussed above*), high *O*-GlcNAc levels, and in some cases increased expression of OGT (140). In sum, elevated protein *O*-GlcNAcylation and OGT expression have been reported in numerous malignancies including breast (16, 63, 64, 141, 142), prostate (143–145), lung (146), pancreas (147), liver (148), and colon (146, 149, 150) cancers. Importantly, levels of *O*-GlcNAc, OGT, and OGA have correlated with aggressiveness (e.g., Gleason score for prostate cancer) in a number of patient tumor samples including prostate (144), breast (64), endometrial (151), and bladder (152) cancers.

One important class of proteins heavily *O*-GlcNAcylated are TFs (Figure 2C). Early analyses suggested that over 25% of known *O*-GlcNAcylated proteins were TFs (14). For many of these TFs, *O*-GlcNAcylation serves as a direct or indirect competitor of key phosphorylation sites (140). Particularly relevant to EMT is the *O*-GlcNAcylation and regulation of SNAI1. Upon serial phosphorylation by CK1 and glycogen synthase kinase (GSK)-3β, SNAI1 is primed for nuclear export, β-TrCP ubiquitination, and subsequent proteosomal degradation (153, 154). Interestingly, SNAI1 is *O*-GlcNAcylated in hyperglycemic conditions preventing GSK-3β phosphorylation, which results in SNAI1 stabilization (155). *O*-GlcNAcylated SNAI1 is associated with enhanced EMT and migration, which is linked to a repression of E-cadherin. Whether other EMT-inducing TFs are similarly regulated by *O*-GlcNAcylation is yet to be determined. Beyond SNAI1, *O*-GlcNAcylation occurs on other TFs generally relevant to cancer including c-Myc (156, 157), β-catenin (158), C/EBPβ (159), p53 (160), and FoxO1 (161), NF-kB (162, 163). Thus, while more experimentation is needed to demonstrate causality between EMT and *O*-GlcNAcylation, *O*-GlcNAcylation has demonstrated to be a key regulator of cancer biology.

Previous studies from our lab and others have elucidated the role of the EMT TF, TWIST1, in suppression of oncogene-induced senescence (OIS) (20, 21, 164). While normal cells respond to oncogene activation with p53-p19^ARF^, p16-Rb, and Atf4-p27^KIP^-dependent OIS (165, 166), suppression of these pathways through EMT TFs provide an alternative route for cancer to maintain cell cycle progression and proceed along a tumorigenic path. Due to the metabolic regulation of the cell cycle, it is not surprising many of these proteins orchestrating cellular division are also *O*-GlcNAcylated. Knockdown of OGT results in elevated expression of p27^Kip^ (63), a reduction of cyclin D1 and B1, and diminished PI3K/AKT signaling (167), suggesting that OGT/*O*-GlcNAc plays key roles during cell cycle progression. Furthermore, OGT is thought to control cytokinesis as it is localized to the mitotic spindle where it interacts with Polo-like kinase. Disrupting *O*-GlcNAcylation results in defects in cytokinesis and multinucleated cells (168). Overall, global *O*-GlcNAc levels have numerous effects on the cell cycle, indicative of yet another link to advancing the neoplastic phenotype.

## Conclusion

The data discussed here highlight alterations in intracellular and extracellular glycoconjugates that impact different EMT tumorigenic pathways and associated proteins/biomolecules. With recent controversies of EMT transcription programs continuing to unfold (169, 170), it is likely that the role of EMT may extend beyond cancer development and metastasis, including cancer treatment resistance. Thus, understanding how changes in metabolic pathways observed in cancer (e.g., the HBP) impact the distribution and composition of glycoconjugates may provide deeper insights into mechanisms of cancer biology. While most of the research discussed here demonstrates the potential for glycoconjugates to regulate EMT, it may be interesting to see in the future how EMT reciprocally promotes metabolic reprograming and the HBP.

## Author Contributions

KT drafted the manuscript and figures based on discussions with Drs NZ and PT. Drs PT and NZ reviewed the manuscript for accuracy and provided constructive criticism while editing the manuscript for flow and content.

## Conflict of Interest Statement

The authors declare that the research was conducted in the absence of any commercial or financial relationships that could be construed as a potential conflict of interest.
